# Protective Effect of Proanthocyanidins from Sea Buckthorn (*Hippophae Rhamnoides* L.) Seed against Visible Light-Induced Retinal Degeneration *in Vivo*

**DOI:** 10.3390/nu8050245

**Published:** 2016-05-02

**Authors:** Yong Wang, Liang Zhao, Yazhen Huo, Feng Zhou, Wei Wu, Feng Lu, Xue Yang, Xiaoxuan Guo, Peng Chen, Qianchun Deng, Baoping Ji

**Affiliations:** 1 Beijing Key Laboratory of Functional Food from Plant Resources, College of Food Science & Nutritional Engineering, China Agricultural University, Beijing 100083, China; WangYongyffs@gmail.com (Y.W.); liangzhao@cau.edu.cn (L.Z.); huoyazhen@gmail.com (Y.H.); bjwfyz_zf@163.com (F.Z.); 18201041642@163.com (F.L.); 13001994248@163.com (X.Y.); guoxiaoxuan083@sina.com (X.G.); 2 College of Engineering, China Agricultural University, Beijing 100083, China; wuwei70@126.com; 3 Oil Crops Research Institute, Chinese Academy of Agricultural Sciences, Wuhan 430062, China; 15827145056@163.com; 4 Hubei Key Laboratory of Lipid Chemistry and Nutrition, Wuhan 430062, China

**Keywords:** proanthocyanidins, sea buckthorn seed, photoreceptor, RPE cells, retinoprotection, visible light-induced retinal degeneration, pigmented rabbit

## Abstract

Dietary proanthocyanidins (PACs) as health-protective agents have become an important area of human nutrition research because of their potent bioactivities. We investigated the retinoprotective effects of PACs from sea buckthorn (*Hippophae rhamnoides* L.) seed against visible light-induced retinal degeneration *in vivo*. Pigmented rabbits were orally administered sea buckthorn seed PACs (50 and 100 mg/kg/day) for 14 consecutive days of pre-illumination and seven consecutive days of post-illumination. Retinal function was quantified via electroretinography 7 days after light exposure. Retinal damage was evaluated by measuring the thickness of the full-thickness retina and outer nuclear layer 7 days after light exposure. Sea buckthorn seed PACs significantly attenuated the destruction of electroretinograms and maintained the retinal structure. Increased retinal photooxidative damage was expressed by the depletion of glutathione peroxidase and catalase activities, the decrease of total antioxidant capacity level and the increase of malondialdehyde level. Light exposure induced a significant increase of inflammatory cytokines (IL-1β, TNF-α and IL-6) and angiogenesis (VEGF) levels in retina. Light exposure upregulated the expression of pro-apoptotic proteins Bax and caspase-3 and downregulated the expression of anti-apoptotic protein Bcl-2. However, sea buckthorn seed PACs ameliorated these changes induced by light exposure. Sea buckthorn seed PACs mediated the protective effect against light-induced retinal degeneration via antioxidant, anti-inflammatory and antiapoptotic mechanisms.

## 1. Introduction

Given high oxygen tension, large doses of visible light exposure and the high proportion of PUFAs in the retina, the retina is particularly susceptible to surrounding light injury and is thus a primary target of photooxidative damage in the eye [1]. A2E, a retinal pigment epithelial (RPE) lipofuscin constituent, mediates light damage because of its capability to absorb photons of specific energy by electron excitation [2]. A2E undergoes photooxidation-induced degradation at sites of singlet molecular oxygen addition, thereby releasing methylglyoxal and glyoxal with the capacity to form advanced glycation end (AGE) products [3]. Visible light can cause damage to the photoreceptors and RPE cells [4,5]. Photoreceptors require photopigments to absorb photons as the first step in seeing; thus, photoreceptors are much more likely to be damaged by excess amounts of visible light [6]. The RPE cells contain light absorbers, such as melanin, lipofuscin and retinoids, which make them susceptible to photochemical damage [6]. Epidemiological studies suggest that photooxidative damage contributes to the onset and the progression of age-related macular degeneration (AMD); furthermore, supplementation with dietary antioxidants might slow the progression of this disease [7,8,9]. At present, AMD is the leading cause of visual impairment and blindness in elderly patients and costs many billions of dollars worldwide [8,10].

Sea buckthorn, a unique and valuable plant that has recently gained worldwide attention, is known as a functional food for its medicinal and nutritional potential [11,12]. When supplementing dry extracts, the range of 500–2000 mg is used for both the berry and leaf extracts. For the oil, slightly high dosage ranges (2000–5000 mg) are used daily [13]. A sea buckthorn seed contains a substantial amount of proanthocyanidins (PACs) [14]. PACs, a group of abundant natural phenolic compounds in plants, have elicited increasing attention in the fields of nutrition and medicine because they have been reported to assume various bioactivities, such as antioxidant [15], anti-diabetic [16], anti-obesity [17], anticancer [18], anti-inflammatory [19] and cardioprotective activities [20]. Adults in the U.S. had a total PAC intake of 95 mg per day [21]. In addition, PACs have been shown to modulate the expression of apoptotic-related genes, to reduce the generation of free radicals and to increase the activity of antioxidant enzymes *in vivo* and *in vitro* [22]. Grape seed PAC extract provided significantly better scavenging activity toward biochemically-generated superoxide anion, when compared to vitamins C and E. PACs are effective protective agents against oxidative damage induced by UV radiation on human keratinocytes and epidermis [23].

PACs can prevent eye malformation [24] and inhibit cataract formation *in vivo* [25]. PACs can prevent oxidative stress-induced damage to retinal ganglion cells by inhibiting apoptotic processes [26,27] and prevent the progression of cataract formation by their antioxidative action [28,29]. PACs notably inhibited AGE formation and significantly reduced dilation of high glucose-induced hyaloid retinal vessels in a diabetic zebrafish model [30]. PACs from lingonberry extract exert protective effects against blue light-induced damage to retinal photoreceptor cells *in vitro* [31]. However, the possibility that sea buckthorn seed PACs could prove beneficial in the amelioration of visible light-induced retinal degeneration has not yet been explored. Based on these reports, we hypothesized that sea buckthorn seed PACs may protect retinal cells against visible light damage.

Previous studies on eye health related to PACs mostly focused on grape seed PACs, which can prevent retinal degeneration *in vivo* and *in vitro*. However, the composition and structure of PACs in sea buckthorn seed and grape seed are different. Fan *et al.* have found that the polymers of sea buckthorn seed PACs are highly heterogeneous; catechin, epicatechin, gallocatechin and epigallocatechin are all constituent components of both the extension and the terminating units [32]. Only B-type PACs were found, and the mean degree of polymerization was 12.2 [32,33]. The proportion of prodelphinidins was 81.2%. Grape seed PAC extract contains approximately 75%–80% oligomeric PACs and 3%–5% monomeric PACs [34]. Catechin, epicatechin and epicatechin gallate are the degradation products of grape seed [35]. Epicatechin is the major component in the extended chain, and catechin is more abundant in terminal units than in extension units [35]. The possibility that sea buckthorn seed PACs can prove beneficial in the amelioration of visible light-induced retinal degeneration has yet to be explored. Therefore, the present study investigated the protective effects of sea buckthorn seed PACs against visible light-induced retinal degeneration *in vivo*. Electroretinography and retinal structure were performed 7 d after light exposure (18,000 lux for 2 h). The antioxidant and anti-inflammatory properties of sea buckthorn seed PACs were analyzed using this animal model. The expression of apoptotic proteins in retinal cells was also measured.

## 2. Materials and Methods

### 2.1. Chemicals

Sea buckthorn seed PAC extract containing 38.9% PACs was provided by Oil Crops Research Institute (Wuhan, China). Tropicamide eye drops were purchased from Xingqi Pharmaceuticals Co., Ltd. (Shenyang, China), sumianxin from Shengda Pharmaceuticals Co., Ltd. (Dunhua, China), anti-caspase-3 from Abcam (Cambridge, UK), anti-Bcl-2 from Abnova (Taipei, Taiwan), anti-Bax from Enzo Life Sciences (New York, NY, USA) and anti-*β*-actin from Cell Signaling Technology (Danvers, MA, USA). Radio immunoprecipitation assay (RIPA) buffer and a bicinchoninic acid (BCA) protein assay kit were procured from Beyotime Institute of Biotechnology (Shanghai, China). All other chemicals and reagents were purchased from Sigma–Aldrich (St. Louis, MO, USA).

### 2.2. Animal Care

A total of 32 healthy pigmented rabbits weighing 2.5–3.0 kg were purchased from the Animal Center of Beijing Kaiyuan Co. (Beijing, China). All procedures were performed in accordance with the Association for Research in Vision and Ophthalmology Statement for Use of Animals in Ophthalmic and Vision Research. The procedures were approved by the Ethical Committee for Animal Experimentation of the First Hospital Affiliated to the General Hospital of the Chinese People’s Liberation Army. All rabbits were housed in a 12 h light–dark cycle for 1 week at 22–25 °C and 55%–60% humidity. All rabbits were fed freely with a standard maintenance diet (Beijing KEAO XIELI FEED Co. Ltd., Beijing, China).

### 2.3. Treatment with Proanthocyanidins from Sea Buckthorn Seed and Exposure to Visible Light

After a week-long adaptation period, the rabbits were randomly divided into four groups (*n* = 8 per group): control group (no light exposure and vehicle administration; CG), light-induced retinal damage model group (18,000 lux light exposure and vehicle administration; MG), treatment group 1 (18,000 lux light exposure and administration of a low dosage of sea buckthorn seed PACs, 50 mg/kg/day; LPACs) and treatment group 2 (18,000 lux light exposure and administration of a high dosage of sea buckthorn seed PACs, 100 mg/kg/day; HPACs). The prescribed dosages of sea buckthorn seed PACs in PBS were intragastrically administered to the rabbits in treatment groups 1 and 2. PBS alone was intragastrically administered to the rabbits in the CG and MG. The rabbits were treated with sea buckthorn seed PACs for two weeks of pre-illumination and one week of post-illumination until sacrifice.

The light exposure method was adopted from our previous study [36]. In brief, after dark adaptation (60–100 lux) for 24 h, the pupils were dilated with tropicamide eye drops at 20 min before light exposure. Non-anesthetized rabbits were placed in cages with a reflective interior and exposed to 18,000 ± 1000 lux from four diffused cool-white fluorescent lights for 2 h. After light exposure, the rabbits were placed in the dark for 24 h and then returned to the normal light/dark cycle.

### 2.4. Electroretinographic Analysis

The electroretinograms (ERGs) were recorded by a visual electrophysiology system (APS-2000AER; Kanghua Rui Ming Technology Co., Ltd., Chongqing, China) 7 days after light exposure as previously described [37]. In brief, after dark adaptation for more than 1 h, the rabbits were anesthetized with an intramuscular injection of sumianxin (0.2 mL/kg) to reduce discomfort. Pupils were fully dilated with tropicamide eye drops. The ERGs were recorded in accordance with the standards set by the International Society for Clinical Electrophysiology of Vision. All procedures were performed under dim red light.

### 2.5. Hematoxylin and Eosin Staining and Measurement at the Full Thickness Retina and Outer Nuclear Layer Thickness

The rabbits were sacrificed after recording the ERGs. HE staining was performed based on the method used in our previous study [36]. In brief, the eyeballs were quickly enucleated and immersed for 48 h in a fixative solution containing 2.5% glutaraldehyde and 2% paraformaldehyde. Samples were embedded in paraffin while considering sample orientation, and 4 μm slides were prepared with a microtome. These sections were prepared with the standard procedure, stained with HE. Micrographs were photographed by a light microscope (Leica, Heidelberg, Germany), and the full thickness retina and outer nuclear layer (ONL) thickness was counted within 250–2750 μm (counted at 500 μm intervals) of the superior and inferior edges to the ONH based on the photographs of HE-stained sections by personnel blinded to the study groups. The mean full thickness retina and ONL thickness were calculated from 12 sections for each retina.

### 2.6. Determination of MDA and T-AOC Levels and GSH-Px and CAT Activities

The levels of total antioxidant capacity (T-AOC) and malondialdehyde (MDA) levels, as well as the activities of glutathione peroxidase (GSH-Px) and catalase (CAT) in the retinal homogenate were determined with enzymatic kits purchased from Nanjing Jiancheng Bioengineering Institute (Nanjing, China). The protein concentrations were determined with the BCA protein assay kit.

### 2.7. Determination of TNF-α, IL-1β, IL-6 and VEGF Levels

The TNF-α, IL-1β, IL-6 and VEGF levels in the retina were determined using a commercially available ELISA kit from Keyingmei Biotechnology and Science Inc. (Beijing, China) in accordance with the manufacturer’s instructions. The protein concentrations were determined with the BCA protein assay kit.

### 2.8. Western Blot Analysis

Whole-cell lysates from the retinal extract were prepared for Western blot analysis by sonication on ice in RIPA buffer containing a protease inhibitor cocktail (Roche, Mannheim, Germany), followed by centrifugation at 15,000× *g* for 30 min at 4 °C to collect the supernatant. Western blot analysis was performed as previously described [37]. After the protein concentrations were determined with the BCA protein assay kit, equal aliquots (20–30 μg) of protein samples were applied to 10% sodium dodecyl sulfate polyacrylamide gels and electrophoretically separated. Resolved proteins were electrophoretically transferred to nitrocellulose membranes (Millipore) and blocked with 5% nonfat dry milk for 1 h at room temperature. The membranes were incubated with Bax (1:1000), Bcl-2 (1:20) or β-actin (1:5000) antibodies for 2 h at room temperature and then with the appropriate horseradish peroxidase-conjugated secondary antibody for 2 h at room temperature. The signals were visualized by enhanced chemiluminescence (Fisher/Pierce, Rockford, IL, USA) and then recorded on X-ray films (Eastman Kodak Company, Rochester, NY, USA). The intensities of the protein bands were determined with ImageJ (National Institutes of Health, Bethesda, MD, USA). The band densities of each sample were normalized to the β-actin band.

### 2.9. Immunohistochemistry

The immunohistological staining was performed based on our previous study [36]. Endogenous peroxidase was quenched by freshly prepared 3% H_2_O_2_ with 0.1% sodium azide. The treated sections were placed in antigen retrieval solution (0.01 mol/L citrate buffer, pH 6.0) for 15 min in a microwave oven at 100 °C and 600 W. The samples were blocked in 10% fetal bovine serum in PBS and then incubated at 4 °C overnight in a primary antibody solution of anti-caspase-3 (ab2171, 1/50). After washing with 0.01 M PBS buffer, the samples were incubated with horseradish peroxidase-conjugated secondary antibodies (1/200, Dako) for 60 min at room temperature, developed with 3,3′-diaminobenzide tetrahydrochloride, counterstained with hematoxylin, dehydrated and then mounted. The staining intensity was scored as follows: 0, negative; 1, weak; 2, moderate; and 3, strong. Positivity was quantified based on the percentage of positive cells: 0, <5%; 1, 5%–25%; 2, 25%–50%; 3, 50%–75%; and 4, >75%. The final score was determined by multiplying the intensity and quantity scores, which yielded a range from 0 to 12.

### 2.10. Statistical Analysis

Results are presented as the mean ± standard deviation. Differences between groups were assessed by one-way ANOVA, followed by Tukey’s test. Statistical significance was set at *p* < 0.05. All statistical analyses were performed using Prism 5.0 (GraphPad Software, San Diego, CA, USA).

## 3. Results

### 3.1. Effect of Sea Buckthorn Seed PACs on Visual Function

**Figure 1:**
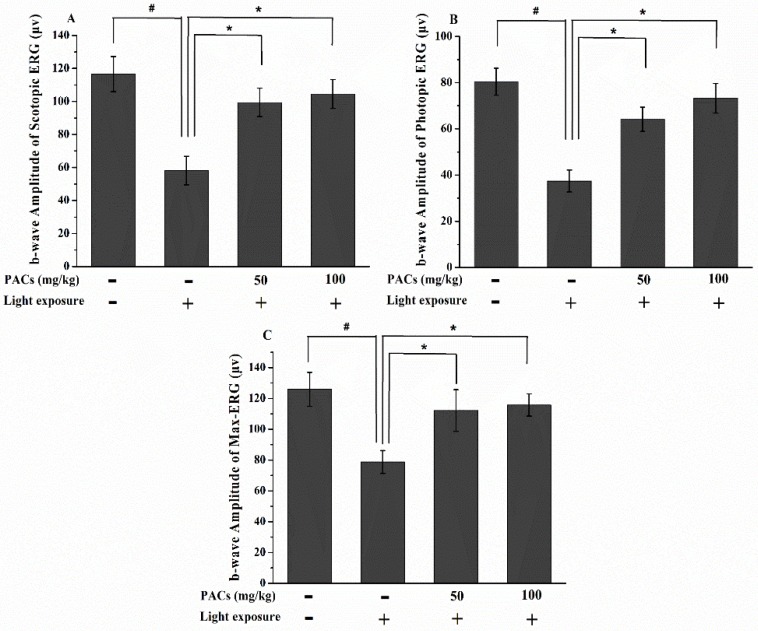
*b*-Wave amplitudes of (**A**) scotopic electroretinogram (ERG), (**B**) photopic ERG and (**C**) max-ERG from retinas in rabbits in the control group (CG), model group (MG), low dosage proanthocyanidins (LPACs) and high dosage PACs (HPACs) groups 7 days after light exposure. Data are expressed as the mean ± standard deviation (*n* = 8). (#, ∗) *p* < 0.05 (one-way ANOVA followed by Tukey’s test). CG: no light exposure and vehicle administration; MG: 18,000 lx light exposure and vehicle administration; LPACs: 18,000 lx light exposure and administration of low-dosage sea buckthorn seed PACs (50 mg/kg); HPACs: 18,000 lx light exposure and administration of high-dosage sea buckthorn seed PACs (100 mg/kg).

In the MG, the *b*-wave amplitudes of scotopic, photopic and max ERG significantly decreased 7 days after light exposure as compared to the retinas in the CG (*p* < 0.05; Figure 1). Compared to MG, the LPAC and HPAC groups showed significantly improved the scotopic, photopic and max ERG function (*p* < 0.05). Thus, sea buckthorn seed PACs supplementation attenuated the damage of photoreceptor cell function in rabbits after visible light exposure.

### 3.2. Effect of Sea Buckthorn Seed PACs on Full Thickness Retina and ONL Thickness

**Figure 2:**
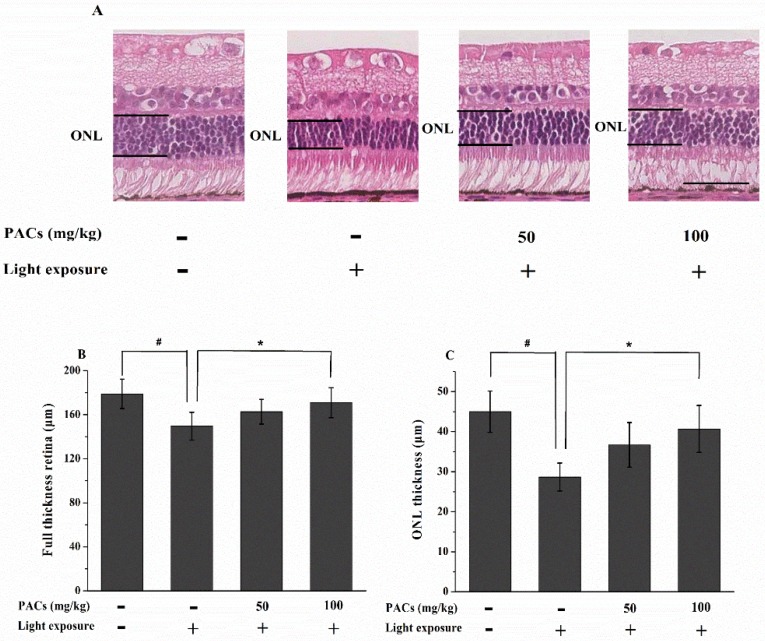
Effect of sea buckthorn seed PACs on the full thickness retina and outer nuclear layer (ONL) thickness in rabbits 7 d after light exposure (18,000 lx). (**A**) Representative images of the HE-stained sections of rabbit retinas. Scale bar: 50 μm. (**B**, **C**) The full thickness retina and ONL thicknesses were measured within 250–2750 μm (counted at 500 μm intervals) of the superior and inferior edges from the ONH based on photographs of HE-stained sections by personnel blinded to the study groups. The full thickness retina and ONL thicknesses were calculated from 12 sections for each retina. Data are expressed as the mean ± standard deviation (*n* = 8). (#, ∗) *p* < 0.05 (one-way ANOVA followed by Tukey’s test).

Considering the protective effect of sea buckthorn seed PACs against light-induced abnormal retinal function, we determined whether the retinal structure was also preserved by sea buckthorn seed PACs treatment. Figure 2 shows the histological analysis of representative retinal images from all study groups in the superior area 7 days after light exposure. The full thickness retina and ONL thickness were remarkably lower in the MG than in the CG (*p* < 0.05), but the decrease in the full thickness retina and ONL thickness were significantly suppressed in the HPACs group compared to the MG (*p* < 0.05).

### 3.3. Effect of Sea Buckthorn Seed PACs on CAT and GSH-Px Activities and MDA and T-AOC Levels

**Figure 3:**
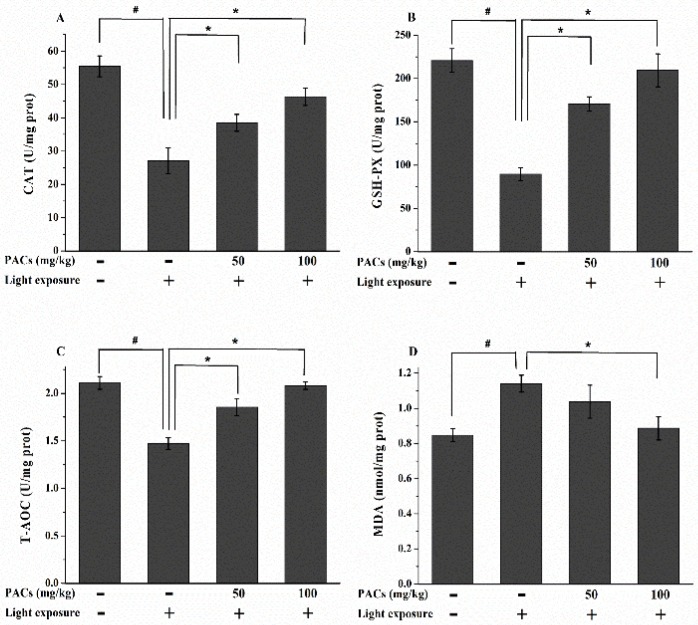
Changes in (**A**) CAT and (**B**) GSH-Px activities, as well as (**C**) total antioxidant capacity (T-AOC) and (**D**) malondialdehyde (MDA) levels, in the retina 7 d after light exposure (18,000 lx). Data are expressed as the mean ± standard deviation (*n* = 8). (#, ∗) *p* < 0.05 (one-way ANOVA followed by Tukey’s test). CAT: catalase; GSH-Px: glutathione peroxidase; T-AOC: total antioxidant capacity; MDA: malondialdehyde.

At 7 day after exposure, the activities of CAT and GSH-Px were significantly lower in the MG than in the CG (*p* < 0.05; Figure 3A,B). However, the activities of these enzymes were significantly higher in the LPAC and HPAC groups than in the MG (*p* < 0.05). The MG showed a significant decrease in T-AOC and a marked elevation in MDA content (compared to the CG, *p* < 0.05; Figure 3C,D), which indicated a decline in antioxidant status. T-AOC significantly increased by 26.53% and 41.50% in the LPAC and HPAC groups, respectively (compared to the MG, *p* < 0.05). The MDA levels of the HPAC rabbits significantly decreased by 21.93% compared to those of the MG rabbits (*p* < 0.05).

### 3.4. Effect of Sea Buckthorn Seed PACs on TNF-α, IL-1β, IL-6 and VEGF Levels

**Figure 4:**
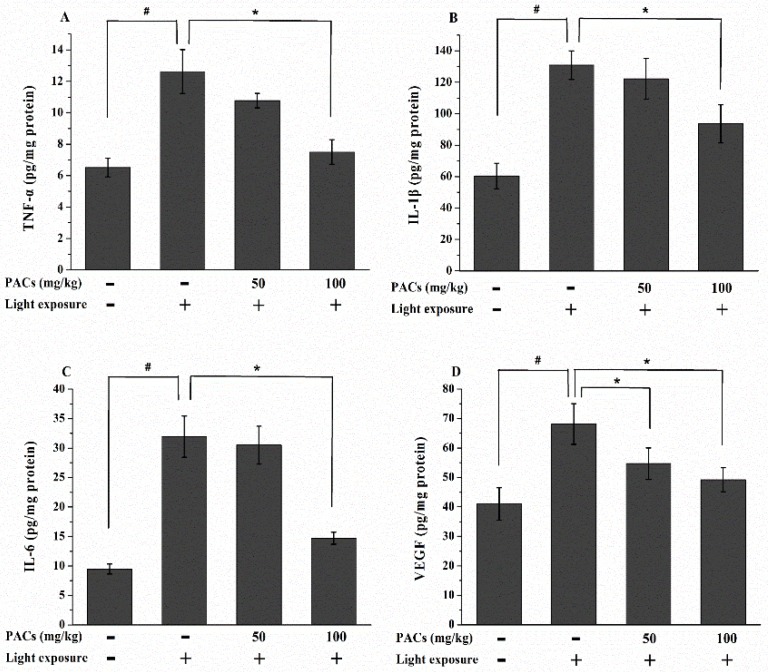
Changes in (**A**) TNF-α, (**B**) IL-1β, (**C**) IL-6 and (**D**) VEGF levels in the retina 7 d after light exposure (18,000 lx). Data are expressed as the mean ± standard deviation (*n* = 8). (#, ∗) *p* < 0.05 (one-way ANOVA followed by Tukey’s test). TNF-α: tumor necrosis factor-α; IL-1β: interleukin-1β; IL-6: interleukin-6; VEGF: vascular endothelial growth factor.

The expression of inflammation- and angiogenesis-related cytokines is associated with retinal photooxidation. Thus, we investigated the levels of TNF-α, IL-1β, IL-6 and VEGF. The exposure of rabbit retinas to visible light increased the secretion of TNF-α, IL-1β, IL-6 and VEGF (*p* < 0.05; Figure 4). However, supplementation with HPACs suppressed the visible light-induced increase in TNF-α, IL-1β and IL-6 secretion (*p* < 0.05). In addition, the VEGF levels of the LPAC and HPAC groups were significantly lower than that of the MG (*p* < 0.05).

### 3.5. Effect of Sea Buckthorn Seed PACs on the Expression of Apoptosis-Related Proteins in the Retina

**Figure 5:**
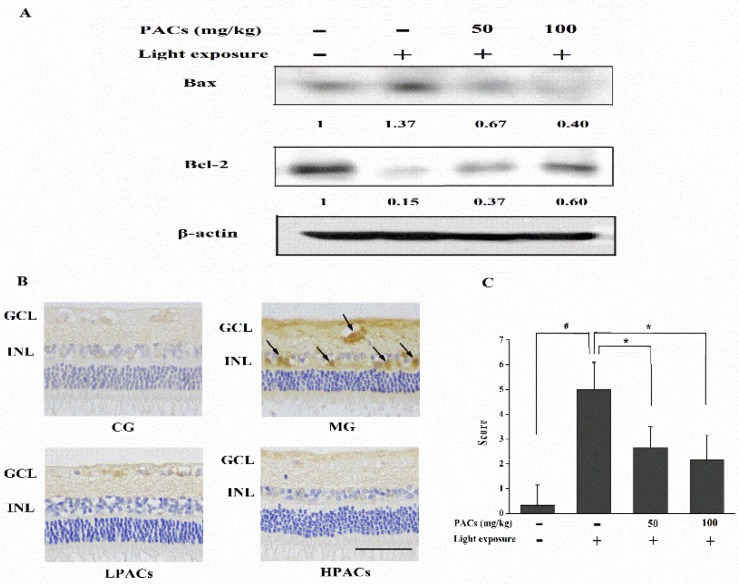
(**A**) Effect of sea buckthorn seed PACs on Bax and Bcl-2 expression in retinas based on Western blot analysis at 7 day after light exposure (18,000 lx). The numbers underneath the blots represent band intensity (normalized to β-Actin loading control, means of three independent experiments) measured by ImageJ software. The standard deviations (all within ±15% of the means) were not indicated. (**B**) Active caspase-3 expression in retinas 7 d after light exposure. Active caspase-3 cells were present, with brown-stained nuclei. GCL: ganglion cell layer, INL: inner nuclear layer. Scale bar: 50 μm. (**C**) Scores were determined by evaluating the extent and intensity of immunopositivity. Data are expressed as the mean ± standard deviation (*n* = 6). (#, ∗) *p* < 0.05 (one-way ANOVA followed by Tukey’s test).

The expression of apoptotic proteins was determined via Western blot analysis or immunohistochemistry. Light exposure upregulated the pro-apoptotic protein Bax and downregulated the anti-apoptotic protein Bcl-2 (Figure 5A). Sea buckthorn seed PACs increased the expression of the anti-apoptotic protein Bcl-2 and decreased the expression of Bax. Caspase-3 activity was not detectable in the CG rabbits (Figure 5B). The expression of active caspase-3 was high in the ganglion cell layer and inner nuclear layer of the rabbit retinas from the MG. However, treatment with sea buckthorn seed PACs reduced the level of active caspase-3 in both layers. The expression of active caspase-3 by immunohistochemistry was scored as shown in Figure 5C.

## 4. Discussion

Supplementation with dietary antioxidants is anticipated to slow the progression of retinal degeneration [38,39]. PACs are the most abundant polyphenols in human diets [40]. Numerous studies have showed that PACs exhibit a wide range of effects, including anti-inflammatory, antioxidative stress, anti-arthritic, anti-cancer and regulation of glycometabolism and lipid metabolism [22]. Considering their anti-oxidative and anti-inflammatory properties, PACs are expected to play a significant role in preventing retinal degeneration. In this study, sea buckthorn seed PACs protected retinal cells from visible light damage *in vivo* and enhanced the antioxidant enzyme activities, inhibiting proinflammatory cytokines, angiogenesis and lipid peroxidation. Protection may occur through these mechanisms.

DHA is the most abundant PUFA in the retina; its high number of double bonds makes it the most easily oxidizable among retinal PUFAs [41]. Light exposure causes the oxidation of retinal DHA *in vivo*, which has been shown to be the main source of reactive oxidation products that induce protein modifications in rod outer segments [42]. Thus, the metabolism and resultant photo-oxidative damage in the photoreceptor cells is extremely high when exposed to high-intensity visible light [43].

Visible light is generated by the Sun, as well as by various artificial illumination sources, such as fluorescent light, light-emitting diodes and monitors of computers, mobile phones and televisions. Studies on photochemical processes suggest that the eye is the most susceptible to visible light and UV radiation [44]. The human retina is protected from high-energy UV light by the cornea and lens, which absorb UV light below 400 nm, but can be damaged by visible light [45]. Therefore, retinal cells, particularly RPE and photoreceptors, are susceptible to injury caused by excessive exposure to visible light, which plays a role in the progression of AMD, as indicated in a human population-based study [46,47]. The prevalence of AMD is estimated to have a one-third increase in the UK and a 50% increase in the U.S. by 2020 [48,49].

PACs, plant polyphenols formed by the polymerization of flvan-3-ols, as cytoprotective agents have become an important source in human health research [50]. The maximum intake of PACs in various human populations can reach 400–450 mg/day [51,52]. The average weight of an adult human is considered 60 kg. The sea buckthorn seed PC extract containing 38.9% PACs was used in this study. Thus, the dose of 50 mg/kg sea buckthorn seed PACs used in rabbits could be considered a moderate dose [53]. A human diet with foods rich in PACs, such as red wine, baking chocolate, apples and dried fruits, could reach dosages up to 700–800 mg/day of PACs [54], which would be about 100 mg/kg in rabbits using the BSA conversion [53]. Thus, 100 mg/kg sea buckthorn seed PACs can be considered a high nutritional dose. In addition, PACs and monomers could be detected in plasma as early as 0.5 h, and the maximal plasma concentration of these compounds was reached at about 2 h after ingestion [55].

Evidence has been presented that TNF-α, IL-1β and IL-6 play an important role in the regulation of inflammatory responses, and those cytokines elevation is associated with many inflammatory disorders [56]. Proangiogenic factors have been shown to be involved in the development of AMD, with VEGF playing the most important role of neovascularization and increasing permeability [57]. NF-κB plays a major role in the regulation of a wide range of genes (TNF-α, IL-1β and IL-6) that are involved in the underlying inflammatory diseases [58]. Epidemiological studies have indicated that populations that consume proanthocyanidin-rich foods have lower incidences of inflammatory diseases [59]. PACs exert potent anti-inflammatory activity through the inhibition of TNF-α, IL-1β, iNOS and COX-2 by regulating the NF-κB and p38 MAPK pathway [60,61]. In this study, sea buckthorn seed PACs decrease the levels of TNF-α, IL-1β and IL-6 in retina of rabbits exposed to visible light.

Human clinical studies show that total plasma antioxidant capacity is increased after oral administration of PACs, which provide a significantly greater protective effect against oxidative damage than vitamin C, vitamin E and β-carotene, because of the powerful antioxidant and free radical scavenging activities [62,63]. In addition, PACs could support indirect antioxidant effects via the regulation of enzymes that inhibit oxidants [64]. PC extract pretreatment maintained the activities of GSH, GPx, SOD and CAT close to normal levels *in vivo* [65]. Grape seed PACs inhibit UV-radiation-induced oxidative stress that inhibited the formation of H_2_O_2_, lipid peroxidation, protein oxidation and DNA damage *in vitro* [66]. In the present study, sea buckthorn seed PACs attenuate the visible light-induced increase in retinal levels of MDA along with a marked increase of CAT and GSH-Px. In addition, the biological effects of PACs are generally attributed to the smaller metabolites (valerolactones, phenylalkyl acids and small aromatics), which are extensively formed and readily absorbed from colonic breakdown of PACs. As the breakdown products of PACs, these metabolites may act as an important contributor to increased plasma antioxidant capacity and exhibit protective effects on retinal degeneration in this study.

## 5. Conclusions

In this study, PACs from sea buckthorn seed possess a protective effect against light-induced retinal degeneration through antioxidant, anti-inflammatory and antiapoptotic mechanisms. Supplementing with PACs may be a useful dietary approach to prevent AMD and other diseases related to retinal photooxidative damage. In addition, the gut-derived microbial metabolites of PACs are the main circulating forms in the blood and accumulation in the retinas. The efficacy of PACs is either via small molecular metabolite(s) and/or mediated by a distant upstream step in the defenses against light-induced retinal degeneration. Therefore, future studies are required to explore which specific forms of PC metabolites are active in the retina.
